# Metformin Preserves Mitochondrial Integrity at Old Age in Male Rats.

**DOI:** 10.1093/geroni/igab046.2576

**Published:** 2021-12-17

**Authors:** Jonathan Wanagat, Allen Herbst, Austin Hoang, Chiye Kim, Judd Aiken, Debbie McKenzie, Deena Goldwater

**Affiliations:** 1 UCLA, Los Angeles, California, United States; 2 University of Alberta, Edmonton, Alberta, Canada; 3 University of Alberta, University of Alberta, Alberta, Canada; 4 UofA, Edmonton, Alberta, Canada

## Abstract

Metformin is being deployed in clinical trials to ameliorate aging in older humans who do not have diabetes. In C. elegans, metformin treatment at old ages exacerbated mitochondrial dysfunction, led to respiratory failure, and shortened lifespan. Metformin is a commonly used, well-tolerated treatment for diabetes in older adults. Mitochondrial effects of metformin treatment in aged mammals has not been sufficiently investigated. We hypothesized that metformin treatment would not be toxic to older mammals. To define a therapeutic dose in aged hybrid rats, we evaluated two doses of metformin (0.1%, 0.75% of the diet) at 30-months of age. Body mass decreased at the 0.75% dose. Neither dose affected mortality between 30- and 34-months of age. We assessed mitochondrial quality, quantity, and function in aged rats treated with metformin at the 0.75% dose by measuring mitochondrial DNA copy number, deletion mutation frequency, and respirometry in skeletal muscle and heart. In skeletal muscle, we observed no effect of metformin on quadriceps mass, mtDNA copy number or deletion frequency. In the heart, metformin treated rats had higher mtDNA copy number, lower cardiac mass and no effect on deletion frequency. Metformin treatment resulted in lower mitochondrial complex I activity in both heart and quadriceps. Metformin did not compromise mitochondrial integrity, was well tolerated, and may have cardiac benefits to rats at old ages.

